# Special Issue Introduction: The Wonders and Mysteries Next Generation Sequencing Technologies Help Reveal

**DOI:** 10.3390/genes9100505

**Published:** 2018-10-18

**Authors:** Manfred G. Grabherr, Bozena Kaminska, Jan Komorowski

**Affiliations:** 1 Department of Medical Biochemistry and Microbiology, Uppsala University, 752 36 Uppsala, Sweden; manfred.grabherr@imbim.uu.se; 2 National Bioinformatics Infrastructure Sweden, Uppsala University, 752 36 Uppsala, Sweden; 3 Department of Cell and Molecular Biology, Uppsala University, 752 36 Uppsala, Sweden; 4 Nencki Institute of Experimental Biology of the Polish Academy of Sciences, 02-093 Warsaw, Poland; b.kaminska@nencki.gov.pl; 5 Affiliated Cancer Hospital & Institute of Guangzhou Medical University, Guangzhou 510095, China; 6 Institute of Computer Science of the Polish Academy of Sciences, 02-093 Warsaw, Poland

The massive increase in computational power over the recent years and wider applications of machine learning methods, coincidental or not, were paralleled by remarkable advances in high-throughput DNA sequencing technologies. Rather than being limited by the number of samples or markers, Life Sciences now face a problem that is well known to information technology: How to mine large data sets for a few signals that are predictive of outcome, in this case phenotype, disease, or responsiveness to medical treatment options. Moreover, data sets are increasingly heterogeneous, capturing both genetic aspects, which might predispose to disease or medical conditions, as well as epigenetic modifications, which can be both inherited and acquired. With millions of data points for hundreds or thousands of samples readily available, research in Life Sciences has turned into the next veritable ‘Big Data’ challenge.

The initial release of the human genome in 2001 [1] came at an expense of 2.7 billion USD [2]. Capillary sequencing, which was used at the time, cost about 1 USD per read (600–900 nucleotides), and the genome build was assembled in a semimanual manner by constructing the sequence of one Bacterial Artificial Chromosome (BAC) [3] at a time. Today, by contrast, standard Illumina protocols and automated computational pipelines produce a complete human genome or methylome for less than 1000 USD, which makes this technology a prime candidate to serve as the backbone for personalized medicine.

Finding the exact genetic variants or changes in epigenetic state that are predictive of disease and disease progression, however, requires a paradigm shift in data analysis that has now started to emerge. Statistical models are increasingly replaced by machine learning (ML) and artificial intelligence (AI), which perform best given large amounts of data and sufficient computational power. Unlike statistical methods, which are restricted to hypothesis testing and produce associations, ML and AI algorithms have the potential to learn new relationships, which can translate into models that are precise predictors for each individual patient. In this Special Issue, the authors report on the state of the art and discuss developments in the near future. 

The successful completion of the Human Genome project and a haplotype map of the human genome provided an incentive for creating large multinational and multi-institutional programs (e.g., the 1000 Genomes Projects [4], Encyclopedia of DNA Elements (ENCODE) [5], and Roadmap Epigenomics [6]) that have the common goal of searching for genomic and epigenomic changes across multiple populations and diseases [7]. Next generation sequencing (NGS) is considered to be a prominent example of a technology generating a huge quantity of Big Data, due to the sheer quantity and enormous complexity of data it produces. It presents an opportunity to use powerful information resources to generate clinically relevant biomarkers or therapy monitoring possibilities, to support better diagnostics and to introduce personalized medicine, which can provide considerable clinical and health economic benefits at a patient and society level.

Personalized medicine includes genetic tests and targeted interventions that are used for a range of purposes, such as risk prediction, treatment decisions, or prenatal screening. This can be focused on either the individual genetic background or the somatic genetic variation that could be acquired, for example, during tumor transformation. The scope of personalized medicine is currently extended to include more than genetic information; in fact, there are attempts to include any disease prevention or treatment approach that takes into account differences in people’s genes, environments, and lifestyle [8]. A certain obstacle to translating NGS into routine health care has been a lack of clinical trials systematically evaluating the performance of NGS technologies, which could allow cost-effectiveness analyses. 

The most common applications of NGS include genomic sequencing, chromatin immunoprecipitation, followed by sequencing of targets (ChIP-seq), RNA sequencing (RNA-seq), methylation site mapping (methyl-seq), and copy number variant detection (CNV-seq). Whole-genome sequencing refers to sequencing the full length of the genome, whereas targeted sequencing allows greater depth of coverage for high resolution analysis of variations within specific genomic regions. Whole-exome sequencing is performed by enrichment in known exons and analysis for genomic alterations. Whole-genome sequencing identifies a fuller spectrum of changes, including structural changes in the genome, such as inversions, deletions, and other genomic rearrangements, in addition to mutations. Transcriptome sequencing (RNA-seq), which has been used routinely for global gene expression profiling, could be used to discover cancer-associated functional gene-fusion transcripts, novel genes or non-coding RNAs. Moreover, development of bioinformatics techniques for ab initio transcriptome assembly led to the discovery of alternatively spliced isoforms and transcripts derived from genomic loci previously considered gene deserts. In 2011, the Illumina BodyMap Project reported as many as 8000 long noncoding RNAs (lncRNA) that are >200 bp in length and are transcribed from intergenic loci that lack protein-coding information. Recent experiments indicate 15,000 long intergenic noncoding RNAs (lincRNAs) encoded by the intergenic regions of human genome. These lincRNAs play important roles in biological processes, such as gene expression control, scaffold formation, and epigenetic control.

A growing area of NGS applications is epigenomics, which extends the epigenetics study from locus and single factors to global analyses of gene regulatory mechanisms. Epigenetics is defined as the “stably heritable phenotype resulting from changes in a chromosome without alterations in the DNA sequence” and comprises different mechanisms, such as DNA methylation, histone modifications, histone variants, nucleosome positioning, and noncoding RNAs (ncRNAs) [9]. Bisulfite conversion of unmethylated C to T, followed by high-throughput sequencing (BS-seq), is a golden standard method to study the methylation status of every cytosine in the genome and produces detailed DNA methylation maps. Popular strategies include Whole-Genome Bisulfite Sequencing (WGBS) and Reduced Representation Bisulfite Sequencing (RRBS). Histone modifications and chromatin-binding factors can be determined by ChIP-seq, in which specific antibodies are used to enrich the DNA fragments at modification/binding sites. At a specific region of the genome, active chromatin is uncondensed, exposes the DNA, and makes it accessible for DNA degradation enzymes such as DNase I and transcriptional machinery. DNase-seq exploits DNase I hypersensitive sites (DHSs) and combines with NGS to uncover the chromatin organization under various conditions. Recently, chromosome conformation capture (3C) techniques have increasingly been used to facilitate the detection of genome folding, chromosome spatial conformation, and long-distance gene–gene interaction. A more advanced Hi-C method is a powerful tool to study genome-wide intra- and interchromosomal interplay, which provides unbiased large-scale information about chromosome structure. 4-cutter Hi-C has been further combined with sequence capture targeting promoters [10] that helps map promoter-enhancer interactions. 

The genomic and transcriptomic composition of an individual cell is lost in conventional NGS studies, which investigate DNA and/or RNA isolated from populations of cells. Some de novo genome mutation and transcriptomic variations in cells could be concealed in the bulk signal. Certain cell types are so rare to detect or isolate with high purity, therefore the application of single-cell approaches becomes necessary for their identification and characterization. Single-cell transcriptomic technology has emerged as a formidable tool to explore cell heterogeneity with a resolution of individual cells. Previous studies of cell biology were limited to data generated by profiling a mixture of cells, frequently in discrete physiological or functional states, which can only provide averaged read-outs that mask cell heterogeneity. This averaging approach is problematic when the biological effect of interest is limited to only a subpopulation of cells within a given tissue or organ [11]. Great advances in single-cell RNA sequencing (scRNA-seq) enabled scientists to overcome this limitation and allow for in depth cross-examination of previously unknown rare cell types. Due to the high sensitivity of scRNA-seq, adequate attention must be put into experimental set-up and execution [12]. 

Genome-wide studies employing NGS and microarrays have shed new light on several areas of Life Sciences. In this issue, the authors investigate the genetic and epigenetic dysfunctions underlying many diseases, but also look into agriculture-related problems. A series of reviews describes applications of abovementioned NGS methods to reveal genetic and epigenetic dysfunctions and alterations in Type 2 Diabetes (T2D), cancer, heart development, and congenital heart disease. Dziewulska et al. [13] summarize applications of NGS technologies to defining the genomic and epigenomic landscape of T2D. T2D is a complex disorder that is caused by a combination of genetic, epigenetic, and environmental factors. High-throughput approaches have provided meaningful insights and opened a new avenue toward a better understanding of the molecular bases of T2D. Barros-Silva et al. [14] focus on high-throughput sequencing techniques that are intensively used in DNA methylation profiling and provide a comprehensive overview of recent advances in using DNA methylation sequencing to find novel biomarkers for detection, diagnosis prognosis, and prediction of response to therapy, as well as to discover new targets for personalized treatments. Pawlak et al. [15] describe the results of comprehensive NGS-based studies within the field of heart biology and pathobiology, where these studies led to the discovery and annotation of novel genetic factors involved in heart development and function. Integrative and comparative NGS-based genomic and epigenomic studies investigate transcriptomic, epigenetic, and genetic determinants of physiological heart development and abnormalities, both in clinics and in model organisms that broaden our understanding. Application of NGS in clinical settings identified a number of genetic factors associated with heart failures. Mustafa et al. [16] successfully validate a target panel-based sequencing of Ion Torrent™ with the Sanger sequencing approach and confirm the suitability of this panel as a first screening test after clinical validation. Finally, Lu et al. [17] move to another domain and investigate the effects of introducing transgenic crops on the soil microbial communities. NGS also facilitates new inroads into agriculture research, an area of importance to society and environment in general. 

The increase in the number of available NGS platforms and further development of machine learning and artificial intelligence-based methods will result in better biomarkers and simpler and faster data analysis pipelines. All these advances will increase the applicability of NGS to basic and clinical studies of different diseases.
